# Evaluation of the effects of berberine in the prevention of epidural fibrosis in rats: An experimental research

**DOI:** 10.15537/smj.2022.43.4.20210918

**Published:** 2022-04

**Authors:** Emrah Keskin, Çağhan Tönge, Mustafa Kaya, Emre Işık

**Affiliations:** *From the Department of Neurosurgery (Keskin), Faculty of Medicine, Zonguldak Bülent Ecevit University, Zonguldak; from the Department of Neurosurgery (Tönge), University of Health Sciences, Gulhane School of Medicine, Diskapi Yildirim Beyazit Training and Research Hospital, Ankara; from the Department of Neurosurgery (Kaya), Sakarya University Education and Research Hospital, Sakarya; and from the Department of Pathology (Işık), Tunceli State Hospital, Tunceli, Turkey.*

**Keywords:** berberine, epidural fibrosis, laminectomy

## Abstract

**Objectives:**

To evaluate the effect of berberine (BBR) in preventing the development of epidural fibrosis (EF) after lumbar surgery in rats.

**Methods:**

This experimental study was carried out at the animal laboratory of Zonguldak Bülent Ecevit University, Zonguldak, Trurkey, between April 2020 and June 2020. A total of 32 Wistar albino female rats underwent laminectomy and were divided into 4 equal groups. Group 1 did not receive any treatment (control group). In group 2, absorbable gelatin sponge was placed at the surgical site. Groups 3 was administered BBR 10 mg/kg and group 4 was administered BBR 60 mg/kg per oral per day for one week after laminectomy. All rats were sacrificed 6 weeks after the operation. Concentration of hydroxyproline (HP) in tissues and histopathological evaluations were carried out to evaluate the level of fibrosis.

**Results:**

Epidural fibrosis results in group 4 were significantly lower than those in groups 1 and 2 (*p*≤0.001). However, there was no significant difference between the mean EF degree between group 2 and group 3. Arachnoidal invasion in both group 3 and group 4 were significantly lower compared to group 1 (*p*<0.05). In terms of HP results, the difference between group 4 and group 1 was statistically significant (*p*<0.001).

**Conclusion:**

This study provides preliminary evidence of the potential use of BBR for preventing the development of EF.

**E**pidural fibrosis (EF) is a potential sequela of laminectomy in patients undergoing spinal surgery. In addition, infection or hematoma may trigger local inflammatory response, aggravating the degree of fibrosis.^1^ Regardless of the cause and degree of fibrosis, the clinical manifestations of traction caused by the scar tissue on the dura and nerve root in the epidural region are referred to as failed back surgery syndrome (FBSS).^2^

Removal of scar tissue in patients who have undergone more than one revision surgery causes additional tissue damage which increases the extent of scar tissue.^3^ Satisfactory results of surgery for EF are obtained only in 1/3 of patients, while 15-20% of these patients experience worsening of symptoms.^4^ Currently, minimally invasive interventions are the mainstay of treatment for EF.^5^ The aim of minimally invasive surgery is to reduce the surgical trauma and bleeding. Applying a drain to the surgical field, autogenous (most commonly fat) or synthetic barrier applications on the dura, and local steroid applications are other preferred applications to reduce EF.^6-8^ Apart from these, the other treatment applications have shown satisfactory results only in experimental studies, and have not yet reached the clinical application stage.^9-11^ Although the aim of both experimental and clinical studies is to minimize fibrosis and prevent FBSS, there is currently no definitive treatment method for EF.

Berberine (BBR) is an alkaloid found in different parts (leaf, stem, root, and bark) of various plants (such as *Berberis vulgaris, Coptis chinensis, Coptidis rhizoma*).^12^ Formulations containing BBR-rich herbal ingredients have been an essential part of traditional Chinese medicine (therapeutic or dietary supplement) for centuries. In the far East, BBR is believed to be a safe and effective remedy for several diseases such as dysentery, inflammation, and hypertension.^13^ Berberine derivatives are used as a fluorescent dyeing method to reveal mast cells, as well as for dyeing skin, wool, and wood.^14^ Several studies have demonstrated the various biological effects of BBR (such as neuroprotective, hypolipidemic, hepatoprotective, antioxidant, and anti-fibrosis) and their underlying mechanisms.^15-19^

In this experimental research, we evaluated the effects of systemic administration of BBR on postoperative spinal EF in a rat model.

## Methods

**Figure 1:**
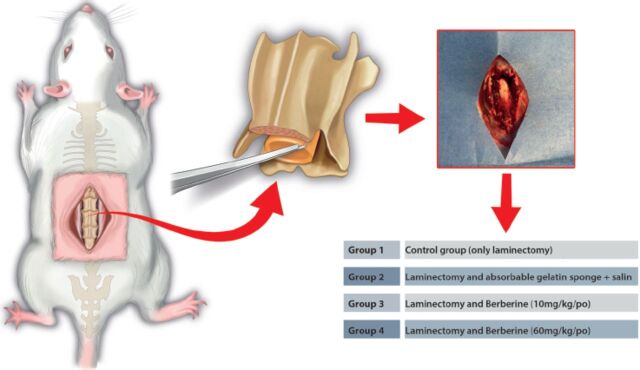
- The experimental design of 4 groups with 8 rats in each group. The dura mater was exposed by laminectomy in all rats in all groups. Low-dose berberine (Group 3) and high-dose berberine (Group 4) were administered to the treatment groups.

This experimental research was carried out on a 12-week-old Wistar albino rats (400±50 grams) between April 2020 and June 2020. The work has been reported in accordance with the Animal Research: Reporting of In Vivo Experiments (ARRIVE) guidelines.^20^ By performing a literature search on Google Academic and PubMed, we reviewed previous studies related to this study. The Ethics Committee approval for this study was obtained from the Bülent Ecevit University, Zonguldak, Turkey, (approval no.: 2020/24/03-09). A total of 32 rats were randomly divided into 4 groups (Figure 1). Sample size was calculated using the resource equation. Accordingly, an E value >20 is sufficient for the study (in this study, E=total number of animals (8×4) - total number of groups [4] = 28).^21^ All groups were housed in separate cages with a randomization similar to that used during grouping. During laboratory follow-up, optimal conditions were provided so that the rats in all groups could easily meet their needs (such as food, drink, and room temperature).

Rats in Group 1 underwent surgery only (control group, no treatment). In Group 2, spongostan (Ethicon, USA, size 2±0.5 mm) was placed in the surgical field after laminectomy. Then, spongostan was soaked with saline (0.5±0.2 mL). Rats in Group 3 (low-dose treatment group) were administered BBR 10 mg/kg/peroral (po), (Sigma-Aldrich, UK), daily for one week after the surgical procedure. In Group 4 (high-dose treatment group) BBR 60 mg/kg/po (Sigma-Aldrich, UK) was administered daily for one week after the surgical procedure.

Surgical procedure was carried out in all groups by the same investigator in the morning at 08:30 until after overnight (12 hours) fasting of rats. Prophylactic antibiotic therapy was provided with intraperitoneal (ip) injection of cefazolin sodium 10 mg/kg (Cefazol; M Nevzat, Istanbul, Turkey), followed by ip xylazine (5 mg/kg; Bioveta, Ankara, Turkey) and ip ketamine hydrochloride (80 mg/kg; Ketalar, Pfizer, Istanbul, Turkey) for general anesthesia. The animals were then placed supine for the surgical position and the surgical site was shaved. Body temperature and respiration were closely monitored throughout the surgical procedure. After cleaning the surgical area (Batticon, ADEKA, Samsun, Turkey), a skin incision corresponding to the L1-L3 distance was made. The muscle and fascia were separated by sharp dissection and the laminae were exposed. Following hemostasis (no cautery was used), total laminectomy was carried out at L1-L3 levels in all groups. After this stage, the dura mater was clearly visible and no cerebrospinal fluid leakage was observed. After all layers were sutured in the anatomical plane, topical antibiotic (Neo-Caf spray, Intervet Productions, Apilia, Italy) was applied to the skin incision line. None of the animals died during or after the surgical procedure. Rats in the treatment groups received po BBR (Group 3 and Group 4) once a day for one week following the surgical procedure. No side effects related to BBR were observed in any of the rats in both groups. On the grounds that bleeding increases fibrosis, spongostan, which is easily absorbable and known to have hemostatic effect, was used in rats in Group 2.^9^ Rats in all groups were sacrificed after 6 weeks of follow-up using overdose of pentobarbital (200 mg/kg; Bioveta, Ankara, Turkey). Except for the skin, bone and all other structures surrounding the surgical field were removed as a single piece as a block. Tissues were transferred to suitable containers for histomorphological and biochemical examination.

The doses of BBR used in this study, low (10 mg/kg) and high doses (60 mg/kg), were based on previous studies.^22^ Relevant concentrations were achieved after dilution with saline.

### Histomorphological evaluation

Tissues were subjected to fixation (7 days) and decalcification (5 days) for 12 days. Sections obtained from the area of the tissue block corresponding to the laminectomy area were embedded in paraffin (3 pieces, 2 mm). Sections were then stained with Masson’s trichrome (Ventana, USA) using an automated stainer (Benchmark, USA). After the staining process, the sections were examined with a microscope (Zeiss Axio Imager 2, Germany) and images were recorded (Zeiss AxioCam ERc 5s, Germany). The results were evaluated in a blinded manner by a pathologist who was unaware of the group identity. Fibrosis scoring was carried out based on the classification system described by He et al^23^ (Table 1). Obtained results were evaluated statistically.

**Table 1:** - Histopathological classification of epidural fibrosis.^23^

Grades	Criteria
Grade 0	No fibrosis affecting the dura mater
Grade I	Only a thin fibrous band between the scar tissue and dura
Grade II	Adhesion continues to progress but does not cover the entire laminectomy defect (less than 2/3)
Grade III	Scar tissue adhesion is larger, constituting more than 2/3 of the laminectomy defect or extending to the nerve root

### Hydroxyproline (HP) colorimetric assay

Tissues were washed with saline and stored under appropriate conditions (-80°C) until tested for HP levels.

Hydroxyproline is one of the most common used markers for evaluation of fibrosis. Hydroxyproline (Hydroxyproline colorimetric assay, acid hydrolysis method, Elabscience, E-BC-K062-S, USA) can produce oxidation product under the action of oxidizing agent. The generated oxidation product reacts with dimethylaminobenzaldehyde to develop a burgundy color. The concentration of HP can be calculated by measuring the optical density value at 550 nm.

### Statistical analysis

Statistical Package for the Social Sciences, version 22.0 (IBM Corp., Armonk, NY, USA) was used. Categorical variables were presented as frequencies and percentages, while the quantitative variables were presented as mean±standard deviation (SD) or median (interquartile range: Q1-Q3). For normal distribution assumptions, since the sample size was <50, Shapiro-Wilk test was used. The Skewness and Kurtosis coefficients and distribution in q-q plot graphs were checked. One Way ANOVA and Kruskal-Wallis tests were used to assess differences among the groups with Scheffe and Tukey post hoc corrections by using the scores depending on the normality of distribution of variables. Between-group differences with respect to categorical variables were assessed using likelihood ratio test. Based on the *p*-values of <0.05 and <0.01, results were considered significant.

## Results

**Figure 2:**
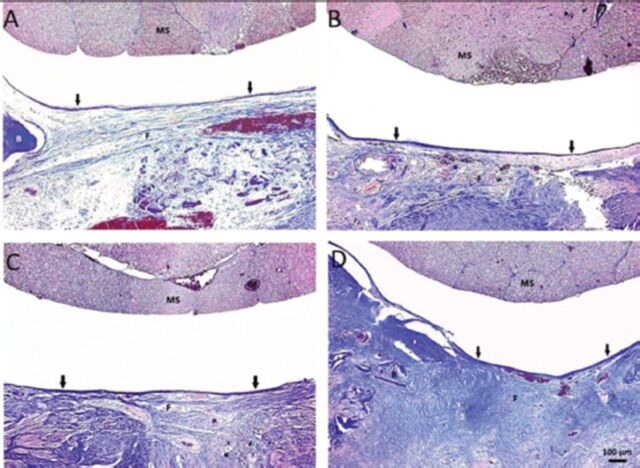
- Histopathological findings (Masson trichrome, original magnification 100×). **A)** Thin epidural fibrosis (F) adhered to dura mater (in group 4, only a thin fibrous band is seen between the scar tissue and the dura). **B)** Epidural F covered less than 2/3 of the laminectomy defect and adhered to dura mater (grade 2 fibrosis in group 3). **C)** Epidural F covered less than 2/3 of the laminectomy defect and adhered to dura mater (grade 2 fibrosis in group 2). **D)** Epidural F completely covered the laminectomy defect and adhered to the underlying dura mater (grade 3 fibrosis in group 1). MS: medulla spinalis, B: bone

Histopathological and biochemical results in all study groups are provided in Tables 2, 3, and 4. The lowest results in terms of EF grades were observed in Group 4 (high-dose BBR), while the highest results were observed in the control (Group 1) and spongostane (Group 2) groups. Epidural fibrosis results in Group 4 were significantly lower than those in the control (Group 1) and spongostane (Group 2) groups (*p*≤0.001). The mean EF grade in the other treatment group (Group 3) was not significantly different from that in the spongostane group (Group 2). Severe EF was observed in 3/4 of the rats in Group 1 (control), while none of the rats in Group 4 (high-dose BBR) showed severe EF (Figure 2). There were significant between-group differences with respect to arachnoidal invasion rates (*p*<0.05). There were no signs of arachnoidal invasion in Group 4 (high-dose BBR), while Group 1 (control) showed the greatest arachnoidal invasion.

**Table 2:** - Comparison of fibrosis grades between the study groups.

Groups	Grade I	Grade II	Grade III	Mean±SD	Groups differences	*P*-value*
		n (%)				
1 (n=8)		2 (25.0)	6 (75.0)	2.75±0.46	*p*<0.01^†^	≤0.001
2 (n=8)		3 (37.5)	5 (62.5)	2.63±0.52	*p*<0.01^‡^	
3 (n=8)	2 (25)	4 (50.0)	2 (25.0)	2.00±0.76	-	
4 (n=8)	3 (37.5)	5 (62.5)		1.63±0.52	*p*<0.01^†^, *p*<0.01^‡^	

Data are presented as number and precentage. One way ANOVA with Tukey post hoc test was used to assess differences between the groups. ^†^Group 1 versus group 4, ^‡^group 2 versus group 4, *comparison of fibrosis grades between groups, group 1: control, group 2: spongostane group, group 3: low-dose berberine (BBR), group 4: high-dose BBR, SD: standard deviation

**Table 3:** - Comparison of arachnoidal involvement between different groups.

Groups	Arachnoidal involvement		*P*-value*
	No	Yes	
	n (%)	
1	3 (37.5)	5 (62.5)	<0.05
2	5 (62.5)	3 (37.5)	
3	8 (100)	-	
4	7 (87.5)	1 (12.5)	

Data are presented as number and precentage. Likelihood ratio test was run for differences among the groups. *comparison of arachnoidal involvement between the groups, group 1: control, group 2: spongostane group, group 3: low-dose berberine (BBR), group 4: high-dose BBR

**Table 4:** - Comparison of hydroxyproline level between different groups.

Groups	n	Median (Q1-Q3)	Groups differences	*P*-value*
1	8	1.54 (1.40-1.77) µ/mL	*p*<0.01†	<0.001*
2	8	1.48 (1.29-1.61) µ/mL	-	
3	8	1.31 (1.24-1.42) µ/mL	-	
4	8	1.19 (0.71-1.33) µ/mL	*p*<0.01^†^	

Kriskal-Wallis with Scheffe post hoc test was used to assess differences between the groups. Results presented as median (interquartile range: Q1-Q3) due to nonparametric distribution. ^†^Group 1 versus group 4, *Comparison of hydroxyproline between groups, group 1: control, group 2: spongostane group, group 3: low-dose berberine (BBR), group 4: high-dose BBR

### Hydroxyproline evaluation

There is a direct relationship between collagen deposition in scar tissue and fibrosis. In this context, HP level, which is considered as one of the markers of collagen accumulation in EF tissue, was measured in all groups (Table 4). There were significant between-group differences with respect to the HP values (*p*<0.001). While the HP level was lowest in Group 4, it was highest in Group 1. The HP level in Group 4 was significantly lower than that in Group 1 (*p*<0.001). In addition, although both low-dose (Group 3) and high-dose (Group 4) treatment groups showed a decrease in HP level, there was no significant difference between these 2 groups (*p*=0.387; *p*>0.05).

## Discussion

Failed back surgery syndrome is defined as the persistence or aggravation of preoperative complaints in patients after undergoing spinal surgery.^3^ Epidural fibrosis, which is among the causes of FBSS, is a physiological side effect seen after all spinal operations, including successful ones.^5^ It is significant that this physiological reaction of the healing process and the term side effect have been used in the same sentence regarding this mostly benign condition. This is because the physiological scar tissue formed during the healing process may cause new clinical symptoms and signs as a result of adhesion and compression of the dura mater and the surrounding nerve tissues over time.^9^

In this study, we examined the effect of BBR, which has been shown to have positive effects on fibrosis in different tissues, in reducing these side effects that may occur due to EF after spinal surgery.

Repeated operations carried out to eliminate the compression effect of EF may lead to increased fibrosis. For this reason, the main goal should be to prevent the development of fibrosis that causes this postoperative result, rather than going from the result to the cause. In this context, many agents have been used in many studies to prevent spinal EF tissue formation, regardless of their biologically inert nature.^6,9-11^ Free adipose tissue has been most frequently used in clinical studies as well as in many experimental studies.^6,24^ However, despite the positive effects mentioned in these studies, the adipose tissue may also cause compression of the spinal nerves.^25^ In our study, BBR, an isoquinoline alkaloid, showed better results histomorphologically (EF and arachnoid invasion) in the high-dose group (Group 4) compared to Groups 1 and 2.

Epidural fibrosis is the result of a physiological cyclical defense reaction during wound healing. In this cycle, there is rapid proliferation of fibroblasts in the healing region under the effect of inflammatory mediators (interleukin-1 [IL-1], IL-6, and IL-8) and growth factors.^26^ Exaggerated healing response leads to increased formation of scar tissue, potentially resulting in EF and causing pressure on the nerve roots. Berberine is widely believed to exert beneficial against many diseases by indigenous people in the far East. Several studies have also demonstrated the neuroprotective and anti-fibrotic of BBR.^16,17,27-29^ Eissa et al^28^ showed that BBR decreased the levels of profibrotic inflammatory mediators and growth factors and increased the level of IL-10 (anti-inflammatory cytokine) in thioacetamide-induced hepatic fibrosis. Based on these results, they hypothesized that BBR suppresses fibrosis secondary to decreased inflammation in liver cells.^28^ Luo et al^29^ showed that BBR prevented apoptosis of human-derived nucleus pulposus cells by reducing oxidative stress secondary to autophagy and endoplasmic reticulum stress due to Ca2+ deregulation. During wound healing, immune cells (such as monocytes and macrophages) together with fibroblasts and smooth muscle cells produce high levels of transforming growth factor beta-1 (TGF-β1).^25^ Transforming growth factor beta-1 further causes proliferation and accumulation of fibroblasts in the extracellular matrix, resulting in a vicious cycle after postlaminectomy EF.^30^ In this context, evaluation of TGF-β1 is important to analyze the anti-fibrotic activity, as it plays a key role among fibrogenic cytokines. Bora et al^31^ showed significant inhibitory effects of both topical and systemic rosuvastatin on EF. They attributed this to the inhibitory effect of rovustatin on the formation of the pro-fibrotic mediator TGF-β1. Bai et al^27^ showed that BBR prevents fibrosis by reducing TGF-β1 production and ultimately reduces the likelihood of lacrimal duct obstruction. In another study, BBR was found to reduce fibrosis by suppressing nuclear factor-kappaB-dependent TGF-β1 expression.^32^ In this context, we believed that the significant inhibitory effect of high-dose BBR on EF observed in our study was mediated via these different pathways in the inflammatory process.

Interleukin-1β is produced by a multiprotein complex called the inflammasome, which its main function is the activation of caspase-1. Node-like receptor protein 3 (NLRP3) is one of these complex family members and is found in the cytoplasm of inflammatory cells.^33^ As a result, IL-1β activated through NLRP3 increases inflammation and ultimately causes fibrosis. Despite the remarkable relationship between NLRP3 inflammation and fibrosis, this relationship has not yet been described in the context of EF. In the study by Jiang et al^34^, BBR was shown to inhibit pro-IL-1β protein, and reduce IL-1β secretion through inhibition of NLRP3 expression. We believed that this potential mechanism of action and positive effect of BBR on neuroinflammation plays a key role in preventing EF.

In this study, Masson trichrome staining was used to assess fibrotic changes, as in previous studies.^29,35^ In this study, histomorphological staging was carried out for EF and arachnoidal invasion using Masson’s trichrome staining 6 weeks after laminectomy.^23^ At this stage, fibrosis was clearly revealed and the results of the staging between all groups were statistically evaluated. Most severe EF was observed in Group 1 (3/4 grade 3, ¼ grade 2). In most rats in this group, EF was observed to extend from the bone defect to the adjacent nerve tissue. Unlike the control group, grade 3 EF was not seen in rats in the high-dose BBR group. In this group of rats, EF was observed in which the scar tissue was limited to the laminectomy defect.

In this study, Masson’s trichrome staining revealed severe fibrosis due to abnormal collagen deposition in Group 1. In addition, we assessed the HP levels, which are considered as biochemical markers of collagen deposition in the wound area during the healing process.^36^ We observed significantly increased HP level in Group 1, which was consistent with the positive association between high HP level and fibrosis. In a study of bleomycin-induced pulmonary fibrosis, Chitra et al^32^ showed that BBR decreased the HP level, an effect that was more pronounced in the early phase (day 14). Similarly, in this study, we observed that Group 4 showed decreased HP level in parallel with our results of Masson’s trichrome. However, HP level was found to be less inhibited in Group 3. The biochemical results suggest that BBR decreased EF.

Severe bleeding may occur during spinal surgery and this play an important role in the development of EF. Although use of passive or active hemostatic agents, electrocautery, and bipolar reduces bleeding, these measures may not prevent the development of fibrosis. We used the passive hemostatic agent Coseal (reabsorbable polyethylene glycol hydrogel) in our previous EF model.^37^ We observed that Coseal decreased EF. However, in this study, Coseal had no significant effect in preventing the development of EF. In this context, although hemostatic agents, electrocautery, and bipolar were not used in our study, BBR reduced the severity of EF in both high- and low-dose groups. In addition to hematoma, wound infection and foreign body reactions may also increase the severity of EF. In this study, no foreign string reaction was observed in the groups treated with BBR (Groups 3 and 4) due to its systemic application. In addition, in these groups, as in the other groups, no side effects (such as wound dehiscence and delayed healing) were observed during the healing process. In the groups treated with BBR, arachnoidal invasion was observed only in one rat in Group 3 and no arachnoidal invasion was observed in Group 4.

### Study limitations

First of all, to support our results, it is important to identify the pathways by which BBR affects EF by in vitro studies. In our study, the results were assessed only up to 6 weeks. Although this period has yielded satisfactory results for assessing EF, longer follow-up may yield more meaningful results. From a different perspective, as reported by Özay et al^38^ in their study on sciatic nerve damage, the surgical area can be opened and closed again at the end of 6 weeks and sacrifice can be carried out at the end of 12 weeks. This may potentially help evaluate the effects of BBR on EF that develops after repeated operations. In addition to including negative control and sham groups in the study, more significant statistical results can be obtained by increasing the number of rats in each group.

In conclusion, this study showed that BBR, which is known to have positive effects on fibrosis in many tissues, can reduce the formation of EF in the laminectomy region. This study provides preliminary evidence supporting the potential benefit of BBR as a new treatment strategy against EF.
